# Predictors of cardiovascular disease among people living with HIV in northern Nigeria

**DOI:** 10.21203/rs.3.rs-8621829/v1

**Published:** 2026-03-08

**Authors:** Zainab Abdulkadir, Aminatu Ayuba Kwaku, AbdulGaffar Lekan Olawumi, Godpower Michael Chinedu, Bukar A. Grema, Fatimah Tsiga-Ahmed Ismail, Baba Maiyaki Musa, William C. Wester, Mahmoud Umar Sani, Muktar Hassan Aliyu

**Affiliations:** Aminu Kano Teaching Hospital; Bayero University Kano; Aminu Kano Teaching Hospital; Aminu Kano Teaching Hospital; Aminu Kano Teaching Hospital; Bayero University Kano; Bayero University Kano; Vanderbilt University Medical Center; Bayero University Kano; Vanderbilt University Medical Center

**Keywords:** Cardiovascular risk, DAD equation, people living with HIV, predictors, prevalence, Nigeria

## Abstract

**Background:**

People living with HIV (PLWH) exhibit two-fold higher incidence of cardiovascular disease compared to HIV-negative persons. However, predictors of cardiovascular disease risk in PLWH are still evolving. The objective of this study is to evaluate the predictors of cardiovascular disease among PLWH in Nigeria.

**Methods:**

This cross-sectional study was conducted among adult patients attending a large HIV clinic in Kano, northern Nigeria. We used systematic sampling to recruit participants and computed their 5-year projected CVD risk using the Data collection on Adverse effects of Anti-HIV Drugs (DAD) equation.

**Results:**

The majority of participants were female (70.6%). The estimated median 5-year CVD risk was 0.7% (interquartile range, IQR 0.4, 10). The majority of participants (58.9%) had a low risk of developing cardiovascular disease, while 28.9% had a moderate risk. Cardiovascular disease was associated with elevated high-sensitivity C-reactive protein (hsCRP) > 3.03 mg/L [adjusted odds ratio, aOR: 4.58, 95% CI: 2.09–10.04), *p* = 0.001], increasing age [aOR 2.38, 95% CI (1.48–4.50), p = 0.020], male sex [aOR 2.16, 95% CI (1.03–4.53), *p* = 0.040] and hypercholesterolemia [aOR 3.03, 95% CI (1.68–4.86), *p* = 0.005].

**Conclusion:**

The majority of PLWH in our setting have low to moderate risk of developing cardiovascular disease. Cardiovascular disease risk was associated with elevated hsCRP, increasing age, male sex, and hypercholesterolemia. Our findings highlight the importance of early CVD risk stratification to prevent morbidity and mortality among PLWH.

## INTRODUCTION

Cardiovascular disease (CVD) is a major global public health concern, responsible for the highest number of deaths worldwide and contributing to over 32% of all deaths in 2023.^1^ People living with HIV (PLWH) are at increased risk of developing CVD.^2^ Despite significant research, the pathophysiology of CVD among PLWH continues to evolve.^3^ The development of CVD in PLWH is due to a complex interplay of traditional CVD risk factors (e.g., hypertension, dyslipidemia, diabetes mellitus, and smoking), HIV-mediated mechanisms, antiretroviral (ARV) medication related cardiometabolic adverse effects, genetic factors, ^4–8^ and increasing life expectancy among PLWH following the widespread availability/access to antiretroviral therapy (ART).^9–10^ HIV itself can induce and hasten atherosclerosis and endothelial dysfunction by several mechanisms, which include chronic inflammation, residual immune activation driven by viral replication, direct effect of the virus on adipose tissue, and altered cholesterol metabolism.^11^ Dyslipidemia, a major CVD risk factor, is highly prevalent among PLWH, with prevalence estimates ranging from 7.7% to 73.4%.^8–10^ This dyslipidemia is attributable to various mechanisms, such as increased basal lipolysis, hepatic de-novo lipogenesis, and hypertriglyceridemia especially following ART initiation.^8,9,10^ In addition, ART exposure may cause fat redistribution, manifesting as lipoatrophy in the face, limbs and viscera, which further exacerbates dyslipidemia and hypertriglyceridemia.^8,9,11,12^

PLWH have double the risk of CVD compared to HIV-negative individuals.^6,8^ Sub-Saharan Africa (SSA) bears the highest burden of HIV globally, with approximately 70% of PLWH residing in the region.^5,6^ However, estimates of absolute CVD risk among PLWH vary significantly across different regions and CVD prediction models.^13–17^ In developed countries, higher CVD risk prevalence among PLWH have been documented.^13–15^ For example, in the United Kingdom, Dhillon et al. reported a prevalence of CVD risk as high as 21.5% using Framingham risk scores (FRS) and 14.8% utilizing the Data Collection on Adverse effects of Anti-HIV Drugs (DAD) risk equation.^13^ Similarly, a study from Serbia reported a prevalence of high CVD risk of 27.2% according to FRS, 31.5% with Systematic Coronary Risk Evaluation (SCORE) and 51.6% using DAD.^14^ In Portugal, high CVD risk was documented at 20.5% using FRS, 10.3% with DAD, and 4.4% with SCORE.^15^

In contrast, studies from developing countries indicate a lower prevalence of CVD risk among PLWH, with regional differences. For example, in Brazil, the prevalence of high CVD risk was reported as 2.8% and 2.1%, using FRS and DAD, respectively. ^18^ In Nigeria, the CVD risk prevalence was reported as 11.7% using FRS, 12.8% using WHO/ISH prediction models, and 12.8% with SCORE.^16–18^ Similarly, an Ethiopian study reported high CVD prevalence among adults aged 20 years and older using FRS and the Pooled Cohort Equations (PCE).^17^ Among PLWH 40 to 79 years of age, PCE yielded higher prevalence (28%) than FRS (17.7%).^17^ In Uganda, the prevalence of high CVD risk was low, at 3.4%, while investigators in Cameroon reported rates of 2.4% utilizing DAD and 8.4% using FRS. ^19–21^ The variations across studies may be due to the use of varying CVD prediction models, the differences in lifestyle and cultural practices between developed and developing countries.

Despite these findings, the prevalence and predictors of CVD among PLWH remain underexplored in northern Nigeria, leaving a critical gap in knowledge. This study aimed to evaluate the prevalence and predictors of CVD among PLWH in Kano, Nigeria.

## METHODS

### Study design and population

This health facility-based cross-sectional study was conducted at the S.S. Wali HIV outpatient Clinic within Aminu Kano Teaching Hospital (AKTH) in Kano, Nigeria. The study utilized data originally collected for a diagnostic accuracy study of hsCRP versus DAD equation for cardiovascular risk assessment.^23^ The current analysis aimed to estimate the prevalence and predictors of cardiovascular disease among PLWH. A total of 180 adults (≥ 18 years of age) living with HIV were systematically recruited over a 6-week period (30th September to 11th November, 2024). We excluded individuals with prior history of cardiovascular events (e.g., known myocardial infarction, stroke, peripheral arterial disease, and/or congestive heart failure), those requiring emergency care, and persons with acute inflammatory illness or moderate to severe cognitive impairment.

### Sampling technique

A total of 180 participants were recruited using systematic sampling, with 10 individuals selected per day using a sampling interval of 5. The first participant was randomly selected through balloting, and every fifth person was subsequently chosen until the minimum sample size was reached.

### Data collection

Data were collected using a pretested questionnaire used in a previous study (available at https://docs.google.com/document/d/1oAEhsbMjn7vNsUTRLj5qyA1lnoxtDqIY/edit?usp=drivesdk&ouid=106491892114506665198&rtpof=true&sd=true).^23^ Trained research assistants administered the questionnaire to obtain sociodemographic information as well as clinical data, including family history of CVD, hypertension, diabetes mellitus, HIV clinical status (CD4 cell count and viral load), medication history (type and duration on ART), weight, height and body mass index (BMI). Blood pressure was measured after 5 minutes rest using a calibrated mercury sphygmomanometer (ERKA)^®^ and an appropriate stethoscope.

Participants were instructed to fast overnight for 8–12 hours, and 5ml of blood was collected into Ethylene-Diamine Tetra-acetic Acid (EDTA) vacutainer tubes for analysis of lipid profile, fasting blood glucose (FBG) and hsCRP levels. Blood samples were transferred to the chemical pathology laboratory on-site at AKTH, where they were centrifuged at 3500 rpm and analyzed further per manufacturer’s instructions. High sensitivity (hs) CRP levels were measured using particle-enhanced turbidimetric assay, calibrated and standardized against the WHO reference values.

Dyslipidemia was classified based on the National Cholesterol Education Program, Adults Treatment Panel (NCEP-ATP) III guidelines.^24^ Hypercholesterolemia was defined as elevated total cholesterol of > 6.2 mmol/L, LDL-C > 4.1mmol/L and/or reduced HDL-C < 1.04mmol/L in men or < 1.29mmol/L in women while, Hypertriglyceridemia was defined as triglycerides of ≥ 1.7mmol/L.^24^ hsCRP cut-off points were obtained after validity testing. Elevated hsCRP was defined as >3.03 mg/L, based on prior research in the area.^23,25^

We defined high blood pressure as systolic blood pressure of ≥ 140mmHg and diastolic blood pressure of ≥ 90mmHg, and/or self-reported history of hypertension or taking antihypertensive medication(s). Diabetes mellitus was defined as fasting blood glucose (FBG) ≥ 7mmol/L and/or self-report or history of taking antidiabetic medication(s). We measured participants’ height with stadiometer (Hospitex)^®^ to the nearest 0.1cm. Weight was measured to the nearest 0.1kg with weighing scale (Hospitex)^®^. We used body mass index (BMI) in kg/m^2^ to classify participants as underweight (< 18.5), normal (18.5–24.9), overweight (25—29.9), and obese (≥ 30) based on WHO guidelines.^26^ Participants’ waist circumference was measured in centimeters at the level of the umbilicus. Using WHO guidelines, truncal obesity was defined as ≥102 cm for men and ≥88 cm in women, while normal waist circumference was <94 cm for men and <80 cm for women.^27^

### Cardiovascular risk assessment

We estimated the 5-year projected CVD risk using the DAD Full (2016) model through a web-based risk calculator.^28^ The calculation included variables such as age, sex, smoking history (past/present), family history of CVD, diabetes mellitus, abacavir exposure, protease inhibitor (PI) exposure and duration, CD4 cell count, systolic blood pressure, total cholesterol, and HDL-C levels. The resulting CVD risk classification was as follows: low risk: <1%, moderate risk: 1–5%, high risk: 5–10%, and very high risk: >10%.^29^

### Statistical Analysis

We analyzed collected data using IBM SPSS Statistical software for windows, version 26. Descriptive statistics were reported as means and standard deviations or medians and interquartile ranges for continuous data, while categorical variables were presented as frequencies and percentages. Associations between categorical variables were tested using chi-square or Fisher’s exact test. Multivariate logistic regression analysis was performed for variables that were significant at bivariate level to identify the predictors of CVD risk. Statistical significance was set at p < 0.05.

### Ethical Considerations

We obtained ethical approval from the AKTH Research Ethics Committee (NHREC/28/01/2020/AKTH/EC/3861). The study adhered to the principles outlined in the Helsinki declaration. Signed informed consent was obtained from all participants.

## RESULTS

The majority of participants were female (70.6%), urban residents (88.3%) and Muslim (85.0%) (Table 1). Most participants reported never smoking cigarettes (91.7%) or consuming alcohol (96.7%). The most common antiretroviral treatment regimen was Tenofovir, Lamivudine and Dolutegravir (TLD), used by 91.7% of participants. The median duration on ART was 12.0 years (IQR: 8.0, 16.0 years). Participants had a mean BMI ± standard deviation (SD) of 24.3 ± 3.9 kg/m^2^, with the majority having normal weight (68.9%), while (13.9%) were overweight and (13.9%) were obese. The mean waist circumference ± SD was 84.9 ± 10.4 cm, and 27.2% of participants presented with abdominal obesity. The most prevalent lipid abnormalities were hypercholesterolemia (25%) and hypertriglyceridemia (19.4%).

Hypertension was observed in 17.8% of participants (classified with JNC-8) while diabetes mellitus was present in 3.9% of participants. Most participants had a CD4 cell count > 200 cells /mm^3^ (86.7%) and undetectable viral load (95.6%), as shown in Table 1.

More than half of the participants (58.9%) were classified as having low cardiovascular risk, while 28.9% were categorized as having moderate risk. Only 12.2% of participants were identified as having a high cardiovascular risk (Figure I). The majority of participants in the low-risk category were women (77.4%) as shown in Table 2.

Tables 3 and 4 depicts factors associated with CVD risk among study participants. Multivariate logistic regression analysis identified age, male sex, hypercholesterolemia, and elevated hsCRP as significant predictors of CVD risk. PLWH who were aged 50 years and older were more than twice as likely to have CVD compared to those under 50 years [adjusted odds ratio, aOR 2.38, 95% confidence interval(CI): 1.48–4.50, *p* = 0.020]. Male participants had more than double the risk of CVD compared to females [aOR 2.16, 95% CI:1.03–4.53, *p* = 0.040].

The majority of participants with high CVD risk had hypercholesterolemia. Hypercholesterolemia remained a significant predictor of CVD after regression analysis. Participants with hypercholesterolemia have more than three times the odds of developing CVD compared to those without hypercholesterolemia [aOR 3.03, 95% CI:1.68–4.86, *p* = 0.005]. Similarly, we found elevated levels of hsCRP > 3.03mg/L to be a significant predictor of high CVD risk after controlling confounders. Participants with elevated hsCRP had more than four-fold higher odds of CVD risk than those with lower hsCRP levels [aOR 4.58, 95% CI:2.09–10.05), *p* < 0.001].

## DISCUSSION

Our study assessed 5-year estimated CVD risk among PLWH in Nigeria, and found the majority of the participants having low to moderate risk. Our findings are consistent with reports from Brazil, Cameroon, and Togo. ^18,20,21^ The observed low to moderate risk of CVD could be attributed to the demographic profile of our participants, most of whom were young females (30–49 years of age). Several studies have reported a low risk of CVD among younger age groups and females.^12,27,29–30^ However, the proportion of participants categorized as high CVD risk in our study (12.2%) was notably higher compared to reports from Brazil (2.1%), Cameroon (2.2%) and Togo (1.5%).^18,20^ This difference may be explained by the longer duration of ART exposure among our study population, with a median of 12 years (IQR: 8, 16 years) compared to median ART durations of 6 years and 4.1 years in the Cameroon and Togo studies, respectively. Additionally, in our study population we documented higher BMI values as 13% of the participants were overweight and additional 13% were obese.^18,20^

The most prevalent traditional cardiovascular risk factors among the study participants were abdominal obesity (27.2%), hypercholesterolemia (25.0%) hypertriglyceridemia (19.4%), and hypertension (17.8%). Diabetes mellitus (3.9%) was the least reported cardiovascular risk factor. These findings are consistent with Noumegni et al in Cameroon, who similarly reported dyslipidemia, abdominal obesity, and hypertension as the most frequent risk factors, while diabetes mellitus being the least prevalent.^20^ The observed prevalence hypercholesterolemia, abdominal obesity, and hypertriglyceridemia could be linked to long-term exposure to ART medications, which are known to induce lipid abnormalities and fat redistribution.^8–11^

Consistent with existing literature, we found older age to be a significant predictor of CVD risk in our population.^31–33^ Male sex was also significantly associated with increased CVD risk, with men exhibiting more than two-fold higher risk compared to women. This finding aligns with studies conducted in the general population and among PLWH.^30–34^ Men faced a heightened CVD risk due to combination of factors, including biological structure and hormonal influences.^35^ For instance, women’s heart and blood vessels are smaller and before menopause estrogen provides women with protection against heart disease.^35,36^ In addition, sex differences in social habits, such as lifestyle choices; smoking, alcohol consumption, and health seeking behavior also contribute to the increased CVD risk among men.^35,36^

Hypercholesterolemia remained a significant predictor of CVD risk in our study, corroborating findings by others. This association may be related to the direct effects of HIV itself on adipose tissue and its impact on cholesterol metabolism.^8–9,11–12^ We also found elevated hsCRP > 3.03mg/L to be a significant predictor of CVD. This finding has been reported by others,^23,25,30^ including Koosha et al who found hCRP biomarker to be an independent risk factor for CVD, independent of age, sex, diabetes mellitus, dyslipidemia, hypertension, obesity, and smoking.^30^

Our study has several limitations. First, we were unable to ascertain causality between the estimated CVD risk and actual cardiovascular events. Second, we cannot generalize our findings to broader populations since this is a tertiary hospital-based study with a limited sample size that may not be representative of all PLWH. Despite these limitations, the study has numerous strengths. We used a globally validated tool for CVD risk estimation specific to PLWH, ensuring accurate risk estimates. In addition, our use of probability sampling minimized selection bias, and regression analysis accounted for potential confounders, strengthening the reliability of our findings.

## CONCLUSION

We found that the majority of PLWH attending a large HIV clinic in northern Nigeria had low to moderate CVD risk. The risk of developing CVD was associated with established risk factors in other studies, namely elevated hsCRP, increasing age, male sex, and hypercholesterolemia. Our findings highlight the importance of early risk stratification and targeted preventive interventions to mitigate the impact of these risk factors among PLWH. We recommend larger, long-term longitudinal studies to better ascertain the incidence of CVD in similar populations and the role of other risk factors, including specific ART regimens and their impact on cardiovascular health.

## Figures and Tables

**Figure 1:**
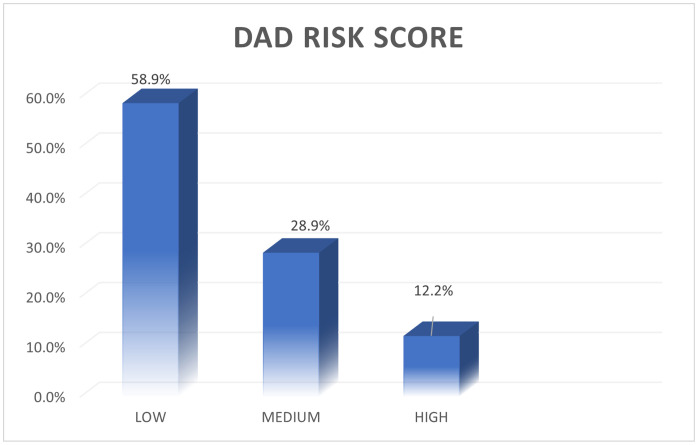
Prevalence of cardiovascular disease risk among the study participants

**Table 1:** Participants’ sociodemographic and clinical characteristics, Kano, Nigeria

Variable	Frequency *N* = 180
Age	
Mean age (years) ±\varvec*S*\varvec*D*	44.03 ± 10.58
Adults 30–49, n (%)	107 (59.4)

Sex	
Female n (%)	127 (70.6)

Place of residence	
Urban n (%)	159 (88.3)

Religion	
Islam n (%)	153 (85.0)

Marital status	
Married n (%)	134 (74.4)

Tribe	
Hausa n (%)	144 (80)

Occupation	
Trader n (%)	38 (21.1)

Level of education	
Secondary n (%)	52 (28.9)

Monthly income (Naira)	
<70,000 n (%)	149 (82.8)

Family history of CVD	16 (8.9)
Present n (%)	

Smoking History	
Never smoked n (%)	165 (91.7)

Alcohol Use	
No, n (%)	174 (96.7)

ART exposure	
1st line, n (%)	165 (91.7)

Median years on ART (IQR)	12 (8,16)

ART Regimen	
2 NRTIs + 1 InSTI (TLD)	170 (94.4)
Protease inhibitors exposed	10 (5.6)

TDF exposed	170 (94.4)

Abacavir Exposed	10 (5.6)
NEV exposed	2 (1.1)
ATV exposed	4 (2.2)

Mean BMI (kg/M^2^) ±\varvec*S*\varvec*D*	24.28±3.90
Normal weight (Kg)n(%)	124 (68.9)
Obesity n(%)	25 (13.9)
Mean waist circumference (cm) ± *SD*	84.91±10.42
Abdominal obesity n%	49 (27.2)

Mean T-Chol(mmol/L) ± SD	5.11±1.19
Triglyceride(mmol/L) ±\varvec*S*\varvec*D*	1.17±0.43,
Mean LDL-C(mmol/L) ±SD	3.38± 1.27
Mean HDL-C(mmol/L) ±SD	1.24±0.37
Hypercholesterolemia n (%)	45 (25.0)
Hypertriglyceridemia n (%)	35 (19.4)

HTN n (%)	23 (17.8%)
Mean systolic blood pressure (mmHg) ±SD	121.67±13.39

Mean diastolic blood pressure (mmHg) ±SD	80.94±7.22

Diabetes mellitus, yes, n (%)	7 (3.9)
Mean FBS mmol/L±SD	4.76±0.86

CD4 count (cells/mm^3^) n(%)	156 (86.7)
≥200 cell/mm^3^	

Viral load (copies/ml) n(%)	172 (95.6)
< 50	

Median DAD Score (IQR)	0.7 (0.4, 10)
Median hsCRP	1.91 (1.4, 2.8)

**Table 2:** Estimated 5-year cardiovascular risk category based on DAD scores by sex, Kano Nigeria

DAD CVD Risk	Male, n (%)	Female, n (%)	Total, n (%)	*P*-value
Low risk (< 1%)	24 (22.6)	82 (77.4)	106 (100.0)	**0.010** *
Moderate risk (1–5%)	17 (32.7)	35 (67.3)	52 (100.0)		
High risk (5–10%)	12 (54.5)	10 (45.5)	22 (100.0)		
Very high (> 10)	-	-	-		

* Statistically significant

**Table 3a:** Participants’ sociodemographic characteristics associated with cardiovascular disease risk by DAD criteria, Kano

Variable	Low (DAD < 1%)	Moderate (DAD 1–5%)	High Risk (DAD 5–10%)	Total	p-value
**Age**					
**<30**	**19(90.5)**	**2(9.5)**	**0(0.0)**	**21(100.0)**	**< 0.001** *
**30–49**	**70(65.2)**	**28(26.2)**	**9(8.4)**	**107(100.0)**	
≥ **50**	**17(32.7)**	**22(42.3)**	**13(25.0)**	**52(100.0)**	

**Sex**					
**Male**	**24(45.3)**	**17(32.1)**	**12(22.6)**	**53(100.0)**	**0.010** *
**Female**	**82(64.6)**	**35(27.6)**	**10(7.9)**	**127(100.0)**	

**Place of residence**					
Urban	94(59.1)	47(29.6)	18(11.3)	159(100.0)	0.57
Rural	12(57.1)	5(23.8)	4(19.1)	21(100.0)	

**Religion**					
Islam	88(57.5)	46(30.1)	19(12.4)	153(100.0)	0.86
Christianity	17(65.4)	6(23.1)	3(11.5)	26(100.0)	
Traditional	1(100.0)	0(0.0)	0(0.0)	1(100.0)	

**Marital status**					0.11
Single	17(85.0)	2(10.0)	1(5.0)	20(100.0)	
Married	76(56.7)	43(32.1)	15(11.2)	134(100.0)	
Divorced /separated	1(100.0)	0(0.0)	0(0.0)	1(100.0)	
Widowed	12(48.0)	7(28.0)	6(24.0)	25(100.0)	

**Tribe**					
Hausa	84(58.3)	43(29.9)	17(11.8)	144(100.0)	0.61
Yoruba	2(66.7)	1(33.3)	0(0.0)	3(100.0)	
Igbo	5(50.0)	2(20.0)	3(30.0)	10(100.0)	
Others	15(65.2)	5(21.7)	3(13.1)	23(100.0)	

**Occupation**					
Civil servant	14(45.2)	11(35.5)	3(9.7)	31(100.0)	0.30
Trader	20(52.6)	3(7.9)	15(39.5)	38(100.0)	
Housewife	45(68.1)	19(28.9)	2(3.0)	66(100.0)	
Farmer	1(25.0)	1(25.0)	2(50.0)	4(100.0)	
Retiree	1(33.3)	2(66.7)	0(0.0)	3(100.0)	
Unemployed	11(84.6)	2(15.4)	0(0.0)	13(100.0)	
Others	14(56.0)	11(44.0)	0(0.0)	25(100.0)	

**Level of Education**					
None	9(50.0)	5(27.8)	4(22.2)	18(100.0)	0.94
Quranic	28(60.7)	13(28.3)	5(10.7)	46(100.0)	
Primary	13(54.2)	8(33.3)	3(12.5)	24(100.0)	
Secondary	33(63.5)	13(25.0)	6(11.5)	52(100.0)	
Tertiary	23(57.5)	13(32.5)	4(10.0)	40(100.0)	

**Income (Naira)**					
<70,000	89(59.7)	44(29.5)	16(10.8)	149(100.0)	0.54
70,000–150,000	14(60.9)	5(21.7)	4(17.4)	23(100.0)	
>150,00	3(37.5)	3(37.5)	2(25.0)	8(100.0	

**Family Hx CVD**					
Absent	102(62.2)	45(27.4)	17(10.4)	164(100.0)	0.14
Present	4(25.0)	7(43.7)	5(31.3)	16(100.0)	

**Smoking History**					
Never smoked	102(61.8)	50(30.3)	13(7.9)	165(100.0)	0.18
Current smoker	3(23.1)	1(7.7)	9(69.2)	13(100.0)	
Past smoker	1(50.0)	1(50.0)	0(0.0)	2(100.0)	

**Alcohol Use**					
No	104(59.8)	49(28.2)	21(12.0)	174(100.0)	0.42
Yes	2(33.3)	3(50.0)	1(16.7)	6(100.0)	

* Statistically significant

**Table 3b:** Participants’ clinical characteristics associated with cardiovascular disease risk by DAD criteria, Kano, Nigeria.

Variable	Low (DAD < 1%)	Moderate (DAD 1–5%)	High Risk (DAD 5–10%)	Total	p-value

ART Regimen	100(58.8)	50(29.4)	20(11.8)	170(100.0)	< 0.001*
2 NRTIs + 1nSTI (TDF exposed)	6(60.0)	2(20.0)	2(20.0)	10(100.0)	
2 NRTIs + 1 PIs	102(60.0)	51(30.0)	17(10.0)	170(100.0)	**< 0.001** *
PIs exposed	4(40.0)	1(10.0)	5(50.0)	10(100.0)	

2NRTIs + ABV	103(60.6)	51(30.0)	16(9.4)	170(100.0)	**< 0.001** *
Abacavir Exposed	3(30.0)	1(10.0)	6(60.0)	10(100.0)	

**Duration on ART**					
< 5yrs	21(75.0)	4(14.3)	3(10.7)	28(100.0)	**0.007** *
≥ 5yrs	85(55.9)	48(31.6)	19(12.5)	152(100.0)	

**CD4 (cells/mm^3^)**					
< 200	10(41.7)	8(33.3)	6(25.0)	24(100.0)	0.071
≥ 200	96(61.5)	44(28.2)	16(10.3)	156(100.0)	

**Viral load (copies/ml)**					
< 50	104(60.5)	50(29.1)	18(10.4)	172(100.0)	**0.045** *
≥50	2(25.0)	2(25.0)	4(50.0)	8(100.0)	

**Comorbidities**					
• HTN	5(15.6)	14(43.8)	13(40.6)	32(100.0)	0.98
• DM	1(14.3)	2(28.6)	4(57.1)	7(100.0)	
• HTN/DM	1(33.3)	0(0.0)	2(66.7)	3(100.0)	
• HTN/Asthma	0(0.0)	0(0.0)	1(100.0)	1(100.0)	
• Asthma	1(50.0)	1(50.0)	0(0.0)	2(100.0)	
• Cancer	0(0.0)	1(100.0)	0(0.0)	1(100.0)	
• Hepatitis B	1(50.0)	0(0.0)	1(50.0)	2(100.0)	
• Absent.	97(71.3)	38(28.0)	1(0.7)	136(100.0)	

**BMI Kg/M^2^**					**0.005**
Underweight < 18.5.	5(83.3)	1(16.7)	0(0.0)	6(100.0)	
Normal ;18.5–24.9	77(62.1)	38(30.6)	9(7.3)	124(100.0)	
Overweight; 25–29.9	16(64.0)	6(24.0)	3(12.0)	25(100.0)	
Obesity ≥ 30	8(32.0)	7(28.0)	10(40.0)	25(100.0)	

**Abdominal obesity**					
Present	24(49.0)	16(32.7)	9(18.3)	49(100.0)	0.48
Absent	72(55.0)	46(53.1)	13(9.9)	131(100.0)	

**Hypercholesterolemia**					
Present	18(40.0)	13(28.9)	14(31.1)	45(100.0)	**0.003**
Absent	88(65.2)	39(28.9)	8(5.9)	135(100.0)	

**Hypertriglyceridemia**					
Present	18(51.4)	13(37.2)	4(11.4)	35(100.0)	0.089
Absent	88(60.7)	39(26.9)	18(12.4)	145(100.0)	

**High-sensitivity CRP**					
Low ≤3.03mg/L	101(70.1)	38(26.3)	5(3.6)	144(100.0)	**< 0.001** *
High > 3.03mg/L	5(13.9)	14(38.9)	17(47.2)	36(100.0)	

* Statistically significant

**Table 4:** Logistic regression analysis showing factors associated with DAD CVD risk, Kano Nigeria

Variables	aOR	OR (95%CI)	P value
Age(yrs)			
< 50yrs	1	(1.48–4.50)	**0.020** *
≥ 50**yrs**	2.38		

Gender			
Female	1	(1.03–4.53)	**0.040** *
Male	2.17		

TDF			
Exposed	1	(0.07–10.74)	0.892
Unexposed	0.84		

Abacavir			
Exposed	1	(0.08–10.99)	0.151
Unexposed	0.91		

Proteases inhibitors			
Exposed	1	(0.09–14.99)	0.189
unexposed	0.89		

ART duration			
≥5yrs	1	(0.16 – 1.32)	0.511
< 5yrs	0.46		

Viral Load (copies/ml)			
< 50	1	(0.03–1.76)	0.159
≥ 50	0.22		

BMI (Kg/M^2^)	1	(0.42–2.10)	0.592
≥30	0.94		
< 30			

Hypercholesterolemia			
No	1	(1.68–4.86)	**0.005** *
Yes	3.03		

hsCRP(mg/L)			
≤3.03	1	(2.09–10.05)	**< 0.001** *
>3.03	4.58		

* **Statistically significant, OR**- Odds ratio, **CI**- Confidence Interval.

## Data Availability

The data set used for this study is available upon reasonable request. The data is not publicly available due to privacy reasons.

## Abbreviations

ART: Antiretroviral therapy
CVD: Cardiovascular-disease
CVR: Cardiovascular Risk
DAD: Data collection on adverse effects of anti-HIV drugs
HIV: Human immunodeficiency syndrome
hsCRP: High-sensitivity C-reactive protein
PCE: Pooled cohort equation
PLWH: People living with HIV/AIDS
SCORE: Systematic coronary risk evaluation
